# The addition of mycoprotein to a mixed-meal impacts postprandial glucose kinetics without altering blood glucose concentrations: a randomised controlled trial

**DOI:** 10.1038/s41430-024-01470-4

**Published:** 2024-07-13

**Authors:** Gráinne Whelehan, Sam West, Doaa R. Abdelrahman, Andrew J. Murton, Tim J. A. Finnigan, Benjamin T. Wall, Francis B. Stephens

**Affiliations:** 1 Department of Public Health and Sport Sciences, Faculty of Health and Life Sciences, University of Exeter, Exeter, EX1 2LU UK; 2 Department of Surgery, University of Texas Medical Branch, Galveston, TX USA; 3 Sealy Center of Aging, University of Texas Medical Branch, Galveston, TX USA; 4 New Era Foods, Hutton Rudby, Yarm, TS15 0JZ UK

**Keywords:** Mass spectrometry, Homeostasis, Type 2 diabetes

## Abstract

**Background:**

Mycoprotein is a high-fibre food previously shown to reduce postprandial glucose concentrations when ingested within a mixed-meal. We applied a dual stable isotope tracer approach to determine whether this is due to a reduced rate of appearance of glucose, in participants of ranging BMI.

**Methods:**

Twenty-four adults (F = 8, BMI 30 ± 6 kg·m^−2^) attended 2 trials in a double-blind, randomised, cross-over design. Participants ingested two energy and macronutrient matched milk-based drinks (enriched with 1000 mg [U-^13^C_6_] glucose in a subset of 12 participants), containing 50 g glucose and either 0 (CON) or 20 g (MYC) mycoprotein. A primed continuous intravenous infusion of D-[6,6-^2^H_2_] glucose determined plasma glucose kinetics over 6 h. Postprandial time-course, and AUC, of glucose and insulin concentration, rate of disappearance (RdT) and appearance of exogenous (RaEx), endogenous (EGP), and total (RaT) plasma glucose were assessed using two- and one-way ANOVA.

**Results:**

Drink ingestion increased blood glucose and serum insulin concentrations (*P* < 0.05) and were comparable between conditions (*P* > 0.05). Both RaT and RdT were higher with MYC compared with CON over 6 h (mean 6 h glucose appearance and disappearance increased by 5 and 9%, respectively, *P* < 0.05). RaEx was not affected by MYC ingestion over 6 h (*P* > 0.05). The mean contribution of EGP to total glucose appearance was 15% greater with MYC, with a trend towards significance (*P* = 0.05). There was no relationship between BMI and the response to MYC ingestion for any of the variables (*P* < 0.05).

**Conclusion:**

The ingestion of mycoprotein within a mixed-meal impacted postprandial glucose kinetics, but not blood glucose or serum insulin concentrations, in individuals of ranging BMI.

**Clinical trial registry number and website:**

This trial was registered at clinicaltrials.gov as NCT04084639 and can be accessed at https://clinicaltrials.gov/ct2/show/NCT04084639.

## Introduction

Repeated exposure to elevated postprandial glucose (PPG) concentrations is a risk factor for the development of Type 2 diabetes mellitus (T2D) [1]. The rate of postprandial glucose appearance (RaT), and the rate of postprandial glucose disappearance (RdT), determine PPG concentrations, and it is often an impairment in RaT and/or RdT that cause elevated PPG. Dietary strategies can be applied to manage such impaired postprandial glucose kinetics. Evidence from several dietary intervention studies, such as the STOP-NIDDM trial, demonstrate that a reduction in PPG by 15–20% has clinically meaningful effects on risk of T2D (i.e., delayed progression to T2D) in people with impaired glucose tolerance [2]. This reduction has been achieved in acute dietary intervention studies by altering the fibre content of a meal. Boers et al. reported a 26% reduction in PPG over 2 h with the addition of 4 g soluble guar gum to a flatbread meal [3], attributed to a lower RaT, due to a small decrease in the rate appearance of exogenous glucose (RaEx), and a greater suppression of endogenous glucose production (EGP) [3]. Nazare et al. also demonstrated a reduced postprandial RaT over 2 h after ingestion of polenta with 5 g of added soluble β-glucan, and this was predominantly due to a reduction in RaEx, rather than EGP [4]. RaT then increased from 2 to 4 h suggesting a delayed rather than reduced rate of glucose absorption, paired with a continued suppression of EGP [4]. Taken together, it would appear that the addition of fibre to a meal can have a profound effect on PPG lasting several hours that is dependent upon the type and amount of fibre.

Mycoprotein is a high-fibre (25 g per 100 g dry weight [dw]) food derived from *Fusarium venenatum*, which contains a unique, predominantly insoluble fibre composition of approximately 2/3 β-glucan and 1/3 chitin [5]. Early studies from Turnbull et al. demonstrated reduced PPG and postprandial insulinaemia (PPI) over 2 h with 20 g dw mycoprotein (i.e. 5 g fibre) consumed during a mixed-meal compared to a low fibre (1 g) macronutrient matched control in healthy young adults [6]. A more recent study by Bottin et al. also reported reduced PPI, but not PPG, over 3 h in overweight adults, with a 40 g dw mycoprotein meal versus a macronutrient matched chicken meal [7]. However, neither of these studies discerned whether this effect was attributable to alterations in RaT (i.e., RaEx and EGP) or RdT.

In this study we used a stable isotope glucose tracer to determine RaT and RdT, to reveal the mechanisms underlying the attenuated PPG response to mycoprotein ingestion. In a subset of participants, we applied a dual stable isotope approach to determine whether an altered RaT may be attributed to changes in RaEx or EGP. We used similar study drinks as provided by Turnbull et al. [6], and we extended the postprandial period to 6 h to identify any delayed effects after mycoprotein ingestion. In addition, we recruited participants across a range of BMI values to determine whether the previously observed attenuation of PPG with mycoprotein ingestion, and the associated alterations in postprandial glucose kinetics, may be greater in those at an increased risk of T2D (i.e. those with a higher BMI) [8, 9]. We hypothesised that the attenuated PPG response observed with mycoprotein would be due to a reduction in RaT from this fibre-rich food.

## Methods

### Participants

Twenty-four adults (females *n* = 8) participated in the present study (CONSORT diagram provided in Supplementary Information Figure S1). Participant characteristics are given in Table 1. Ethical approval was obtained from University of Exeter Sport and Health Sciences Ethics Committee (190206/B/06). Each participant provided informed consent for the study. The trial was registered on clinicaltrials.gov as NCT04084639. This study was conducted in accordance with the Declaration of Helsinki. Recruitment and data collection were completed between April 2019 and November 2021.

**Table 1:** Participant characteristics.

Female/Male, *n*	8/16
Age, y^*^	30.1 (8.4)
Height, cm	173.2 (9.5)
Weight, kg	92.0 (21.5)
BMI, kg٠m^−2^	30.5 (5.7)
Fasting Glucose, mmol٠L^−1^	4.6 (0.4)
Fasting Insulin, pmol٠L^−1^	96.1 (48.2)
HOMA-IR	2.9 (1.6)

^*^Age at screening.

Data presented as mean (SD).

HOMA-IR is homeostasis model assessment-insulin resistance.

### Experimental protocol

This study used a double-blind cross-over design. Treatment allocations were balanced according to a Williams-type design, and a randomised schedule for allocation to treatment orders was generated by software randomizer.org.

Participants attended an initial screening visit followed by 2 experimental visits at the University of Exeter’s Nutritional Physiology Research Unit laboratories, with a washout period of at least 2 weeks in between. To standardise the participants’ basal state, participants were advised to minimise changes in their habitual diet and activity, while avoiding foods naturally enriched in ^13^C such as maize, pineapple, tropical fruits, canned foods, for 3 days before each trial. The participants refrained from alcohol consumption and vigorous activity 24 h before each trial and followed a standardised meal-plan the day before each visit providing 50–55% energy from carbohydrates, in accordance with procedures for standardising glucose tolerance testing [10]. All participants fasted overnight but could drink water ad libitum.

The participants arrived at the laboratory at 0700 ± 1 h following a 10 h overnight fast. The participant rested in a semi-supine position and a cannula was inserted in the forearm for infusion of [6,6-^2^H_2_] glucose tracer. Another cannula was inserted in a retrograde fashion into a dorsal hand vein and placed in a heated hand-box (55°) to obtain arterialised venous blood samples. At *t* = −120 min, a priming dose of [6,6-^2^H_2_] glucose (80 × 0.07 mg‧kg^−1^ body weight) was administered as a bolus in 60 mL of 0.9% saline in 2.4 min at a rate of 1500 mL‧min^−1^, followed immediately by a continuous infusion of 0.07 mg‧kg^−1^ for 8 h. At *t* = 0 the participant ingested the drink within 5 min. For the initial 12 participants recruited, 100 mg of [U-^13^C_6_] glucose (enrichment of 0.16%) was added to test drinks to determine RaEx and EGP. However, after initial analysis of plasma samples for [U-^13^C_6_] glucose it was clear that the enrichment was not high enough above background enrichment to sensitively measure RaEx i.e., RaEx did not return to baseline after several hours (Supplementary Information Fig. S2). Thus, a subset of participants (*n* = 12) received test-drinks containing 1000 mg [U-^13^C_6_] glucose (enrichment of 1.6%) in order to calculate RaEx.

### Sample collection

Before starting the infusion, two venous blood samples were taken 5 min apart for measurement of background enrichment of [6,6-^2^H_2_] glucose and [U-^13^C_6_] glucose in plasma. Arterialised venous blood was subsequently sampled at −30, −15, −1 min, and then sequentially every 15 min up to *t* = 120 min post-consumption of the drink and then every 30 min up to *t* = 360 min. Except for the measurement of blood glucose, which was performed immediately (YSI 2300 STAT, Yellow Springs, Ohio), whole blood was dispensed into lithium-heparin tubes and serum-separating tubes, which were mixed by inversion. Lithium-heparin tubes were then centrifuged (4 °C and 3500 *g*) for 10 min to obtain plasma. Serum tubes were left to stand at room temperature for 30 min before centrifugation. The serum and plasma supernatant were then dispensed into 1 mL aliquots and immediately frozen at –20 °C, and transferred to storage at −80 °C at end of test-day until assay.

Rates of carbon dioxide (VCO_2_) production and oxygen (VO_2_) consumption were measured using a metabolic cart (Cortex Metalyzer 3B, Leipzig, Germany) at baseline for 20 min before ingestion of the drink, and then for 10 min every hour following the test drink. The first and final 2.5 min of each measurement were discarded to remove any potential error associated with the early stabilisation of metabolic rate, and any disturbances associated with termination of the measurement. Details of calculations are provided in Supplementary Information. Participants were not permitted to sleep, watch television, read or listen to music during measurements.

### Test drinks

Two milk-based drinks containing 50 g glucose (Dextrose, Bulk Powders^™^, Essex, UK) were produced to enable the study to be conducted blind. The test drinks were prepared by an independent researcher not involved in subsequent analysis. The nutritional contents of the drinks are given in Table 2. The ingredients were matched as closely as possible to those provided by Turnbull [6], but with full-fat dried milk powder added to the control (CON) drink instead of full-fat soya flour to better match total protein and energy content. This also ensured minimal fibre present in the CON condition. The mycoprotein drink (MYC) contained 20 g freeze-dried mycoprotein (Marlow Foods Ltd, Quorn Foods, Stokesley, North Yorkshire, UK).

**Table 2:** Nutritional content of the experimental drinks.

	CON	MYC
Energy, kJ	1995.4	1989.1
Energy, kcal	476.3	475.8
Carbohydrate, g	74.5	74.5
* Glucose, g*	50.0	52.0
* Lactose, g*	24.0	22.0
Fibre, g	0.2	5.0
Protein, g	17.8	17.8
Fat, g	11.9	11.9
* Saturated, g*	7.7	6.6
* Monounsaturated, g*	3.2	2.9
* Polyunsaturated, g*	1.0	2.2

All experimental drinks contained 250 mL full-fat milk and 50 g of dextrose.

CON contained 18.7 g dried skim milk and 9 g full-fat dried milk.

MYC contained 10.7 g lactose and 20 g mycoprotein.

### Sample analysis

Serum insulin concentrations were measured by enzyme immunoassay sandwich technique using a commercially available kit (DRG Insulin ELISA, EIA-2935, DRG International Inc.). Plasma GIP concentrations were determined by RIA, based on an antibody that fully reacts with the primary metabolite GIP3-42 (RayBio®, USA). Plasma concentrations of GLP-1 were analysed by ELISA, which measured the sum of the intact GLP-1 and its primary metabolite GLP-1 9-36 amide (Epitope Diagnostics, Inc. San Diego, USA). All samples were analysed in batches after all experimental trials were completed, and all samples for a given participant (from the two trials) were run on the same plate. Details of plasma kinetics analysis and calculations of both kinetics and insulin sensitivity indices are provided in Supplementary Information.

### Statistical analyses

Effect and sample size were calculated using data from Boers et al. [3], that observed a reduction in total appearance of glucose over 4 h with the ingestion of 10 g fibre (Cohens *d* = 1.5) with 12 participants. For the present study, we halved the effect size based on the 5 g fibre in MYC and the power calculation indicated that 24 participants were required for 80% power to detect a reduction in total appearance of glucose after MYC ingestion compared with CON at a significance level of 0.05. Data are presented as mean (SEM) unless otherwise stated. One-way repeated measures ANOVAs were used to assess differences between the trials at baseline and for summary measures (e.g. AUCs). Two-way repeated measures ANOVAs (time x treatment) were used to identify differences between conditions. In the event of a significant interaction effect post-hoc analysis using Tukey’s multiple comparisons tests was performed. Our primary measure was glucose rate of appearance (RaT). Blood glucose concentrations, serum insulin concentrations, plasma GIP and GLP-1 concentrations, energy expenditure and carbohydrate and fat oxidation represent our secondary measures. For tertiary analyses we also examined whether a correlation existed between BMI and the above listed variables response to MYC.

## Results

### Postprandial glucose and insulin response

**Fig. 1:**
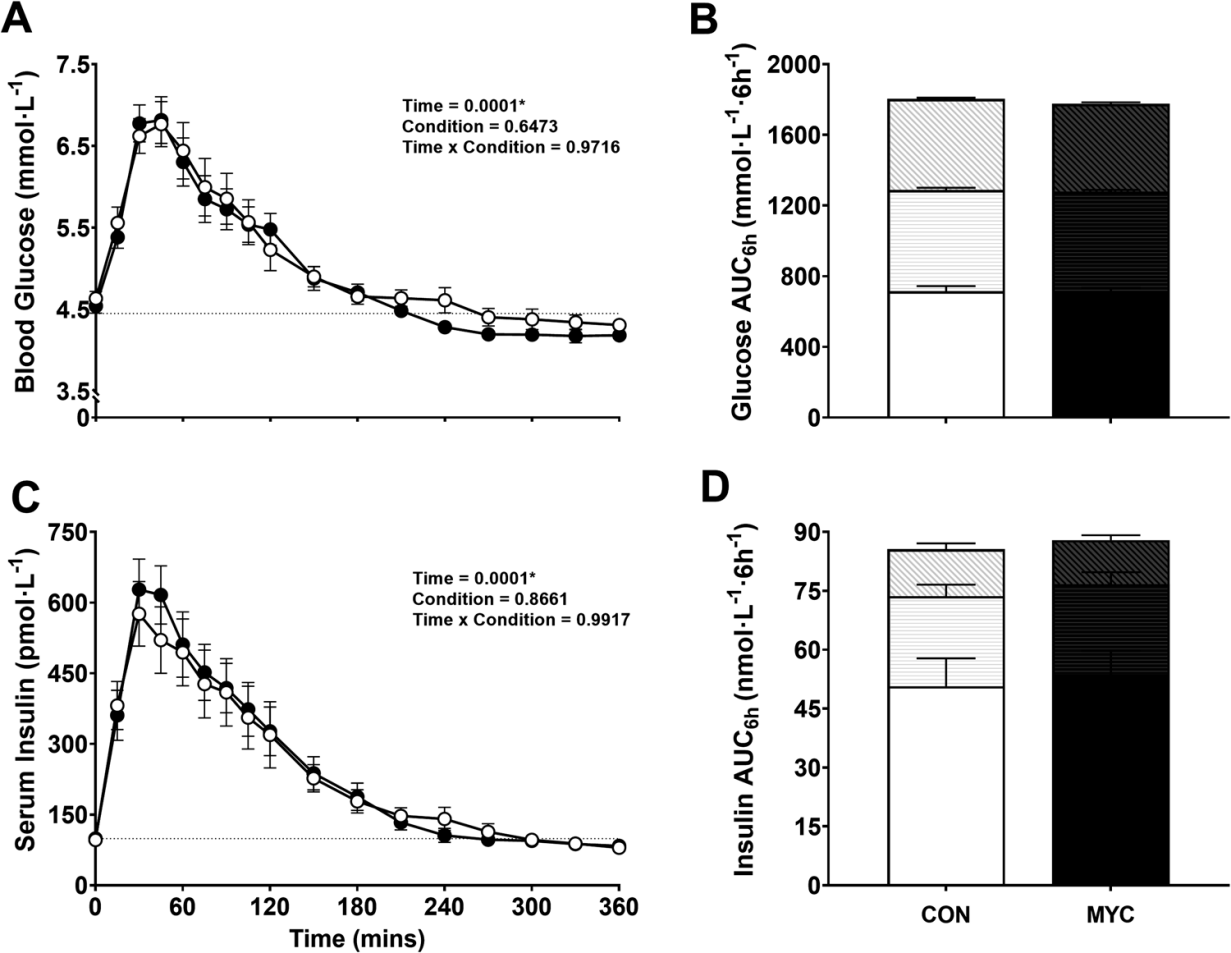
Postprandial blood glucose and serum insulin concentrations. Blood glucose concentrations (**A**), serum insulin concentrations (**C**), AUC for glucose (**B**) and insulin (**D**) over 6 h postprandial following ingestion of the control (CON) and mycoprotein 20 g (MYC) drinks. Gridline depicts mean baseline (fasting) concentrations. 6 h AUC is split into three time epochs; 0–120, 120–240 and 240–360 min, represented by the base colour, horizontal bars and diagonal bars, respectively. Error bars depict SEM. A main effect of time was observed for all conditions in glucose and insulin (*P* < 0.05).

After ingestion of both CON and MYC blood glucose concentrations increased (Fig. 1) (*P* < 0.0001). There was no difference in the glycaemic response between CON and MYC, i.e., both conditions caused a mean peak blood glucose concentration of 7.3 mmol٠L^−1^ at 41 min, with no difference in total AUC over 6 h, mean (SEM); 1804 (42) vs. 1778 (36) mmol٠L^−1^٠6h^−1^ for CON and MYC, respectively (*P* = 0.28), or during the initial 2 h post-ingestion, 720 (29) vs 712 (25) mmol٠L^−1^٠2h^−1^ (*P* = 0.57).

As with blood glucose concentrations, both CON and MYC ingestion caused a significant increase in serum insulin concentrations from baseline (96 [7.5] pmol٠L^−1^) (Fig. 1) (*P* < 0.0001), with no difference over 6 h between conditions. MYC had a greater peak of 722 (59) compared to 667 (66) pmol٠L^−1^ after CON ingestion (*P* = 0.02). Over 6 h, and in the initial 2 h post-ingestion, MYC did not cause a different insulinaemic response (*P* > 0.05). There was no relationship (*P* = 0.14) observed between BMI and PPG, however PPI significantly correlated with BMI (*P* = 0.001, r = 0.6) (data not shown). This relationship did not impact the PPI response to MYC ingestion.

Postabsorptive and postprandial values for blood glucose and serum insulin concentrations were used to estimate insulin sensitivity (Table 3). There was no difference in any of these indices between conditions (*P* > 0.05).

**Table 3:** Insulin sensitivity indices.

	CON	MYC	*P* value
Insulinogenic Index	2.2 (1.5)	2.1 (1.2)	0.626
Matsuda	6.2 (3.3)	5.5 (2.3)	0.080
Cederholm	22.7 (4.4)	22.5 (4.3)	0.649
Disposition Index	11.5 (7.5)	11.5 (9.0)	0.987

### Plasma glucose kinetics

**Fig. 2:**
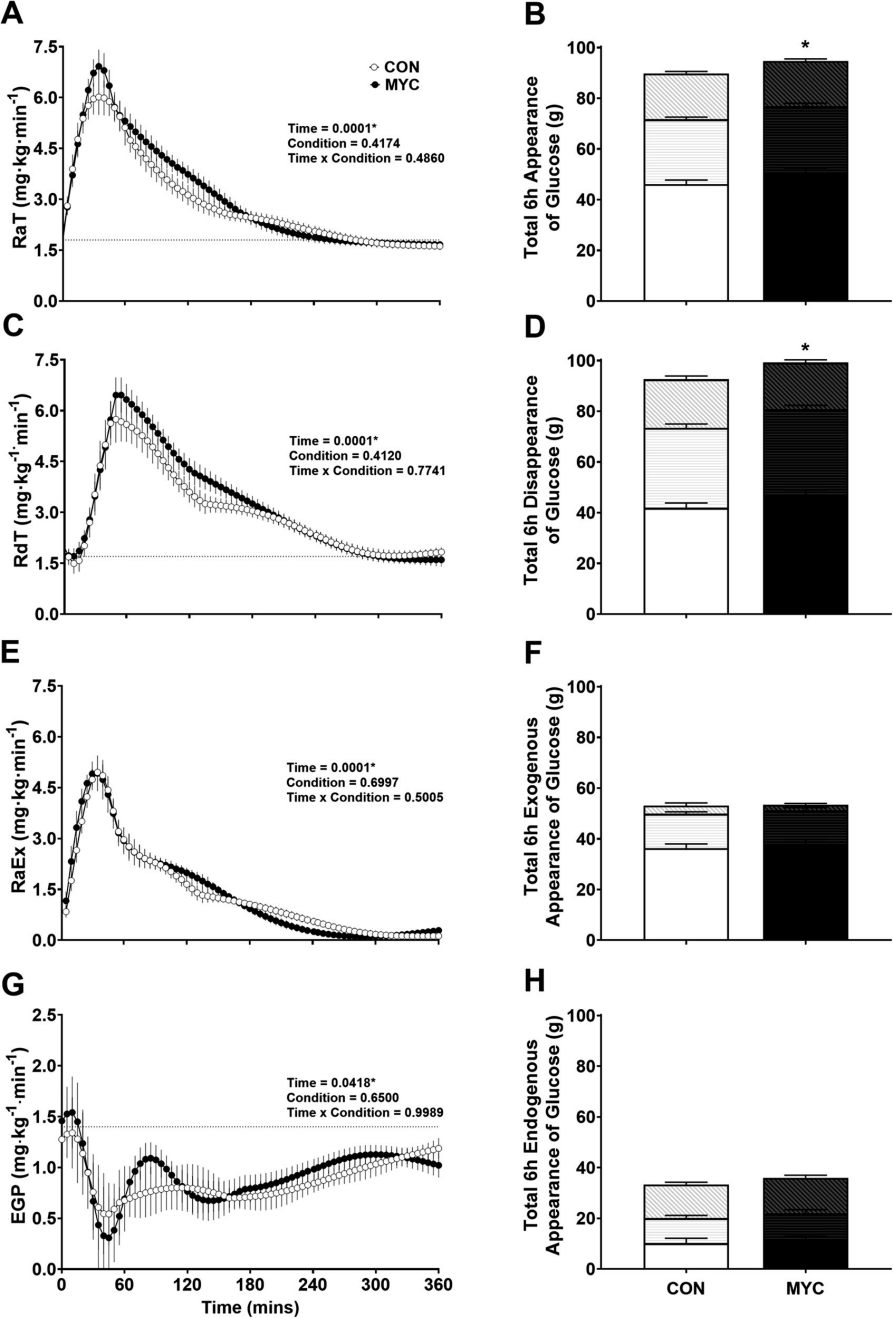
Plasma glucose kinetics following ingestion of the control and mycoprotein drinks. Total rate of glucose appearance (RaT), total rate of disappearance (RdT), total rate of appearance of exogenous glucose (RaEx) and total rate endogenous glucose production (EGP) after ingestion of the control (CON) and mycoprotein (MYC) drinks are shown over 6 h in graphs (**A**, **C**, **E**, **G**). Gridline depicts mean baseline (fasting) values. Appearance and disappearance over 6 h for the above kinetics are displayed in graphs (**B**, **D**, **F**, **H**) and are split into three epochs, as depicted by the base colour, horizontal bars and diagonal bars to represent 0–120, 120–240 and 240–360 min, respectively. Error bars depict SEM. Statistical significance indicated by *P < 0.05.

Both CON and MYC increased RaT and RdT from baseline (*P* < 0.0001). Figure 2 shows the response curves for the smoothed kinetics data including the total amount of appearance and disappearance of glucose over 0–120, 120–240, 240–360 and 0–360 min. RaT did not differ between conditions over 6 h (*P* = 0.50). When calculated as a total appearance of glucose over 6 h, 90 (12) g and 95 (13) g of glucose appeared with ingestion of CON and MYC respectively, i.e., an increase of 5% over 6 h with MYC ingestion (*P* = 0.03). Figure 2 shows that the majority of this glucose appeared within the first 120 min, i.e., 46 and 51 g (51 and 53% of total) for CON and MYC. The overall 6 h greater appearance of glucose in the MYC condition is primarily explained within this first time epoch. RdT did not differ over 6 h with MYC ingestion compared with CON (*P* = 0.77). When calculated as a total disappearance over 6 h, MYC had a significantly greater (7%) disappearance of glucose (99 g) compared to CON (93 g) (*P* = 0.004). As with the appearance of glucose, the overall 6 h differences in disappearance were due to a difference of 5 g within the first 120 min, 42 (9) g and 47 (9) g of glucose disappeared after CON and MYC, respectively.

A subset of participants (*n* = 12) had 1000 mg of ^13^C glucose added to CON and MYC drinks in order to discern between EGP and RaEx, from the total appearance of glucose. Smoothed data are displayed (Fig. 2E–H) and raw data are supplied in Supplementary Information Fig. S3. There was no difference in the appearance of exogenous glucose over 6 h between conditions (*P* = 0.50). Over the 6 h postprandial period, mean contribution to total glucose appearance from exogenous glucose was 62 and 60%, with the primary contribution from exogenous glucose within the first 2 h post-ingestion, contributing a mean of 69 and 71% of total exogenous glucose appearance for CON and MYC, respectively. 53 g appeared in systemic circulation over 6 h for both conditions, i.e., approximately 70% of ingested carbohydrate (or 100% of ingested free glucose). The contributions of endogenous and exogenous glucose to total glucose appearance across the 6 h were equivalent between conditions (Fig. 2F, H). During the first 2 h, the majority of glucose appearance (~77%) comprised exogenous glucose. This then shifted during the subsequent 2 h whereby the contribution of exogenous glucose decreased (to ~60% of total) and endogenous increased (to ~40% of total). The final 2 h results in a further shift resulting in a majority contribution from endogenous glucose (~81%).

### Substrate oxidation

**Fig. 3:**
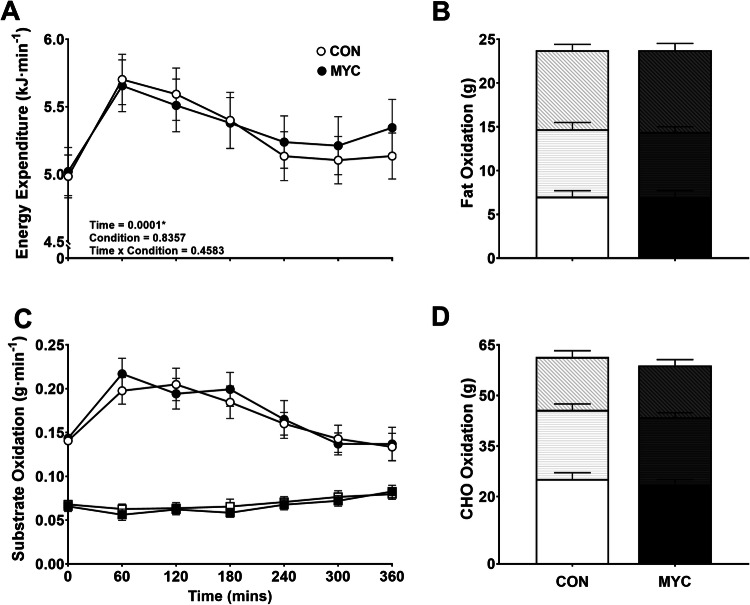
Energy expenditure and substrate oxidation. Energy expenditure (**A**), fat oxidation (C + B) and carbohydrate (CHO) oxidation (C + D) from baseline to 6 h post-ingestion of drinks: control (CON) and mycoprotein (MYC). Error bars depict SEM. Graph **C** shows substrate oxidation of CHO (circles) and fat (squares). Graphs **B** and **D** represent oxidation over 6 h and are split into thirds to represent three different postprandial time-periods; 0–120, 120–240 and 240–360 min. A significant effect of time was observed in all conditions for EE, CHO oxidation and fat oxidation (*P* < 0.0001). There was no significant difference between drinks for EE (*P* = 0.97), CHO oxidation (*P* = 0.52) or fat oxidation (*P* = 0.58).

After ingestion of both CON and MYC energy expenditure (EE) significantly increased from baseline (*P* < 0.0001). Over 6 h, the mean (SEM) of total EEs for CON and MYC were 1920 (292) and 1904 (378) kJ, respectively (*P* = 0.88). Average total carbohydrate (CHO) oxidation over the 6 h postprandial period was 62 (21) and 58 (20) g (*P* = 0.47), and fat oxidation was 24 (9) and 24 (9) g for CON and MYC, respectively (*P* = 0.85) (Fig. 3). The contribution of CHO and fat oxidation to total EE shifted over the 3 time epochs for both conditions. At basal, CHO oxidation contributed 48 and 50% of total EE for CON and MYC. In the first 2 h postprandial, CHO oxidation increased this contribution to 60 and 57%, between 2 and 4 h this decreased to 55 and 54%, and between 4 and 6 h this decreased further to 45 and 46% for the CON and MYC conditions. There were no differences in substrate oxidation between the two conditions.

## Discussion

The aim of the current study was to elucidate the mechanisms behind the attenuated postprandial glucose (PPG) previously observed with the ingestion of mycoprotein within a mixed-meal [6, 7], using a dual stable isotope technique. Due to the high fibre content of mycoprotein we hypothesised that the attenuated glycaemic response would be due to a lower RaT. However, we could not test this hypothesis as the improved glycaemic response with mycoprotein in the aforementioned studies was not observed in the current study. Potential reasons for this are discussed below.

A previous study that provided 20 g mycoprotein within a mixed-meal reported significantly lower postprandial serum glucose concentrations at 30 min compared to a control, and a 7 and 16% reduction in PPG (glucose AUC_0-120_) and PPI (insulin AUC_0-120_), respectively, with the mycoprotein condition [6]. Interestingly, despite the present study using similar drinks to that provided by Turnbull et al., there was no difference in blood glucose or serum insulin concentrations after ingestion of MYC compared with CON. The mycoprotein drinks from both studies are close to identical in ingredients and nutritional composition, yet Turnbull reported a mycoprotein mean glucose AUC_0-120_ of 590, versus 712 mmol‧L^−1^‧2 h for MYC in the present study. It is worth noting, however, that the present study recruited participants across a range of BMI values (22.2-38.5 kg٠m^−2^), which resulted in higher levels of insulin resistance in this group of participants (HOMA-IR 3.7 versus Turnbull 1.6), and insulin resistance was correlated with PPG and PPI response. This explains the higher absolute mean PPI and PPG in the present study. However, this does not explain the lack of an observed effect of mycoprotein on PPG/PPI, i.e., there was no relationship between HOMA-IR/ BMI and the PPG/PPI response to MYC ingestion compared to CON. These differences between studies are difficult to reconcile as the mycoprotein drinks are identical in composition, however, it could be due to differences in composition of the control drinks. The soya flour provided in the control drink in Turnbull’s study was replaced by full-fat dried milk powder in this study, which has a higher carbohydrate and saturated fat content. The type of carbohydrate is also different, with dried milk powder containing 100% lactose, and soya flour with 66/34% of starch/sucrose [11], and this difference in carbohydrate type alone could impact PPG and PPI [12, 13]. Thus, although the control drinks from both the present study and Turnbull’s study were identical in macronutrient composition, it is possible that the nutritional differences in the milk powder and soya flour were too different to be comparable.

The more recent work of Bottin et al. also did not show a difference in PPG with ingestion of a mycoprotein meal compared to a control meal in obese and overweight adults (BMI 25–32 kg٠m^−2^) [7]. However, the test meals were provided as macronutrient-matched ‘risotto’ meals, thus likely not comparable to the present study due to potential differences in digestive efficiency with solid versus liquid meals. The meals also contained ~40 g mycoprotein versus chicken, with much lower amounts of carbohydrate (25 g) and greater amounts of protein (40 g) compared to the present study (75 g carbohydrate and 18 g protein) suggesting that the absence of an effect on glycaemia may be due to insufficient carbohydrate provided. Nevertheless, Bottin et al. did demonstrate a significantly lower insulin concentration for the same PPG, which highlights the importance of measuring the underlying glucose kinetics.

The present study hypothesised that RaT would be decreased with the ingestion of mycoprotein. This hypothesis was based on results from Boers *et al* that demonstrated a significantly lower RaT with the addition of guar gum to a meal compared to a control [3]. However, Boers et al. used 3 types of flat breads with an equal amount of total fibre in each condition, but with a different proportion of soluble and insoluble fibre. As the proportion of soluble fibre (guar gum) increased, the greater the reduction in RaT was observed [3]. The present study hypothesised that the 5 g additional fibre in MYC would reduce RaT, however ingestion of MYC resulted in a 5% increase in total glucose appearance over 6 h, possibly suggesting that the ‘type’ of fibre in mycoprotein may not be appropriate for a reduction in PPG. Typically, it is ‘viscous’ fibres that have the most potent effect on reducing PPG [14] and the type of fibre in mycoprotein is ~88% insoluble [15], which are typically non-viscous and have previously had no impact on RaT [16, 17]. Studies that have observed a reduced PPG with insoluble fibre have administered much higher doses than the present study. For example, Schenk *et al*. [17] compared 35 g versus 0.5 g in breakfast cereals, Weickert et al. provided over 10 g insoluble fibre to reduce PPG [18], and Roberston *et al*. [19] demonstrated the consumption of 60 g resistant starch over 24 h reduced both PPG and PPI. The present study provided ~4.4 g insoluble fibre and it is possible, based on the above studies, that this dose was too small to observe a reduction in PPG, and potentially, RaT.

A previous study that also demonstrated a reduction in PPG and RaT added 5 g β-glucan to a carbohydrate meal (72 g CHO) [4]. The link between β-glucan and reduction of PPG is well-established [20], most likely due to reduced carbohydrate absorption from delayed gastric emptying [21, 22]. Nazare et al. demonstrated a reduction in RaEx by 21% compared to carbohydrate alone in overweight adults [4]. The fibre in mycoprotein is made up of approximately 2/3 β-glucan, and thus approximately 3.3 g β-glucan was provided in MYC. However, the β-glucan linkages in the cell-wall of plants (β1,3 and β1,4) are different to that of fungi (β1,3 and β1,6) [23], and therefore will act different physiologically and cannot be assumed to reduce PPG and RaT (RaEx) by the same mechanism. Indeed, with the addition of the ^13^C oral tracer it revealed an equivalent RaEx between conditions, i.e., the fibre in MYC20 did not delay carbohydrate absorption. Colosimo et al. demonstrated that the cell-wall of mycoprotein can entrap α-amylase and reduce the rate of starch hydrolysis, in vitro [24]. The authors suggest this as a possible mechanism by which mycoprotein could reduce PPG and PPI in vivo. However, the present study provided carbohydrate in the form of free sugars, and thus there is no further breakdown required and a slowing of digestion by the method described above would not be possible.

The presence of mycoprotein caused an increased RdT compared to CON. However, this was balanced by an increased RaT and thus did not modify PPG concentrations. The increase in RdT was accompanied by a greater insulin peak after MYC, although total insulin exposure (AUC) was the same. This earlier insulin peak was also observed by others [17, 18] after intake of insoluble fibre. We recruited participants across a range of BMI values in an attempt to represent a greater spectrum of glucose tolerance, however all of the recruited participants, except one, had a 2 h glucose below 6 mmol٠L^−1^, significantly lower than the cut-off for impaired glucose tolerance of 7.5 mmol٠L^−1^ [25]. This was due to the higher insulin concentrations in those with a higher BMI. Arguably, these participants do not require a further insulin stimulus, as they can maintain normoglycaemia. It would be interesting to determine whether this enhanced insulin peak would occur in those that have lost the first-phase insulin secretory response, as in T2D [26], and whether this would assist in the management of postprandial glucose control. It is worth noting, however, that based on the ‘normal’ glycaemic response observed in all participants, that BMI is likely an imperfect indicator of glycaemic status and to more accurately obtain a spectrum of glucose tolerance (and insulin sensitivity) an oral glucose tolerance test conducted during screening would have been necessary. Other limitations include the greater number of males than females, and it would have been preferable to have an equal number of both to avoid any possible impact of sex on glycaemic outcomes.

Taken together, the dose of insoluble fibre provided in MYC was likely not sufficient to reduce PPG when consumed with free glucose. Investigation into whether higher doses of mycoprotein improve glycaemic control is warranted, particularly based on the results of previous trials that have demonstrated improved glucose tolerance and peripheral insulin sensitivity with daily consumption of high-doses of insoluble fibre.

### Supplementary information

Supplementary Information

## Data Availability

Data described in the manuscript, code book, and analytic code will be made available upon request pending approval by the corresponding author.
