# Supplementary material for: The addition of mycoprotein to a mixed-meal impacts postprandial glucose kinetics without altering blood glucose concentrations: a randomised controlled trial
Source: Eur J Clin Nutr. 2024 Jul 13;78(9):757–64. doi: 10.1038/s41430-024-01470-4 (PMC11368804; doi:10.1038/s41430-024-01470-4)
Supplement: Supplementary file 1 — Supplementary Information [file 41430_2024_1470_MOESM1_ESM.docx]

**Supplementary Information**

**Sample analysis**

To calculate kinetic parameters of RaEx, RaT, EGP and RdT, fractional enrichments of orally and intravenously administered [U-^13^C_6_] glucose and [6,6-^2^H_2_] glucose, respectively, were determined in the collected plasma samples by gas chromatography-mass spectrometry (GC-MS). Samples were deproteinised by adding 300 µL of ice-cold methanol to 50 µL of plasma and placed on ice for 30 min. Following centrifugation for 12 min at 12000 *g* at 4ºC, a volume of 300 µL supernatant was transferred to a glass screw neck vial and air-dried.  Anhydride:pyridine (2:1) was added to the dry samples, vortex mixed, and left at room temperature for 30 min to form a penta-acetate glucose derivative. The samples were then analysed by GC-MS (6890N GC coupled with a 5975B MSD; Agilent Technologies) and in duplicates using electron impact ionisation and selected ion monitoring for measurement of isotope ratios. One µL of the sample was injected in splitless mode (injector temp. 280°C). Peaks were resolved using an HP5-MS 30 m × 0.25 mm ID × 0.25 μm capillary column (Agilent). Helium was used as carrier gas at 1.2 ml‧min^-1^ constant flow rate. The temperature ramp was set from 80 – 260°C at 15°C‧min^-1^.  Selected ion recording conditions were used to monitor fragments m/z 200, 202 and 205 for glucose.

**Calculations**

RaT (mg∙kg^-1^∙min^-1^) (**Eq. 1**) was calculated by using the non-steady state equation of Steele as adapted by De Bodo [1], with a value of 0.23 L∙kg^-1^ for volume of distribution (V) as proposed by Livesey, and a pool fraction value of 1 was used [2]. $F_{\left[ 6,6-^{2}H_{2} \right]-glucose:}$ is the [6,6-^2^H_2_]-glucose infusion rate (0.07 mg∙kg^-1^∙min^-1^), p is the pool fraction (1 L), 180.16 is the molecular weight of glucose (g∙mol^-1^), C_1_ and C_2_ are the glucose concentrations at t_1_ and t_2_ (mmol∙L^-1^), ${IE1}_{[6,6-^{2}H_{2}]-glucose}$ and ${IE2}_{[6,6-^{2}H_{2}]-glucose}$ are the plasma [6,6-^2^H_2_]-glucose isotopic enrichments at t_1_ and t_2,_ respectively (mol % excess (MPE)), and t_1_ and t_2_ are the first and second collection time points (min).

**Eq.1**: $RaT=$

$$\frac{F_{\left[ 6,6-^{2}H_{2} \right]-glucose}-pV \cdot180.16 \cdot\frac{\left( C_{1}+C_{2} \right)}{2}\cdot(\frac{{IE2}_{[6,6-^{2}H_{2}]-glucose}-{IE1}_{[6,6-^{2}H_{2}]-glucose}}{t_{2}-t_{1}})}{(\frac{{IE1}_{\left[ 6,6-^{2}H_{2} \right]-glucose}+{IE2}_{\left[ 6,6-^{2}H_{2} \right]-glucose}}{2})}$$

RaEx (mg∙kg^-1^∙min^-1^) (**Eq. 2**) was calculated using Steele’s equation for non-steady state [3] as adapted by Schneiter, where$\boldsymbol{F}_{\boldsymbol{infusion}}$ is the total glucose perfusion rate (0.07 mg∙kg^-1^∙min^-1^)**,** V is the volume of distribution for glucose (0.23 L∙kg^-1^∙min^-1^), ${IE1}_{[13C_{6}]-glucose}$ and ${IE2}_{[13C_{6}]-glucose}$ are the plasma [$13C_{6}$]-glucose isotopic enrichment at t_1_ and t_2_ (mol % excess (MPE)),[U-^13^C_6_]glucose: is the amount of [U-^13^C_6_]glucose ingested (g) and glucose: is the total amount (labelled and unlabelled) of glucose ingested (g).

**Eq. 2**: $RaE=\frac{{(RaT-F}_{infusion})\cdot(\frac{{IE1}_{[13C_{6}]-glucose}+{IE2}_{[U-13C_{6}]-glucose}}{2})+pV \cdot180.16 \cdot\frac{\left( C_{1}+C_{2} \right)}{2}\cdot(\frac{{IE2}_{[U-13C_{6}]-glucose}-{IE1}_{[U-13C_{6}]-glucose}}{t_{2}-t_{1}})}{(\frac{\left[ U-13C_{6} \right]-glucose}{glucose})}$

Endogenous glucose production (EGP) was calculated as RaT-RaEx. Identical behaviour of labelled and unlabelled glucose molecules was assumed. The baseline sample for each trial was used to account for background isotopic enrichments. After calculating RaT, RdT and RaEx for each time-point, data was then smoothed using Graphpad Prism 9. A cubic spline curve was fitted to each individual’s data between the time periods; 0-60, 60-180 and 280-360 min [4]. The program interpolated at time intervals of 1 min which was used to calculate individual total appearance and disappearance of glucose in grams (0-120, 120-240, 240-360 and 0-360 min). EGP was calculated using the interpolated data from RaT and RaEx. This data was then fitted with a spline curve as described. Unsmoothed curves are presented in the Supplementary Information (see Supplementary Information Figure 3).

Area under the curves (AUC) were calculated as an integrated expression of glucose and insulin concentration over time. Insulin sensitivity indices were calculated according to Matsuda [5], Cederholm [6], Seltzer (Insulinogenic Index) [7] and Bergman (Disposition Index) [8].

Rates of whole-body fat and carbohydrate utilisation were calculated using the equations of Frayn [9]. CHO oxidation (g·min^-1^): $\left( 4.55* {VCO}_{2} \right)-\left( 3.21* {VO}_{2} \right)$ and fat oxidation (g·min^-1^): $\left( 1.67* {VO}_{2} \right)-\left( 1.67* {VCO}_{2} \right)$. Energy expenditure was calculated using the formula of Weir [10], energy expenditure (EE) (kJ·min^-1^): $\left( 4.187 \right)* \left( 3.941* {VO}_{2} \right)+\left( 1.106* {VCO}_{2} \right)$.


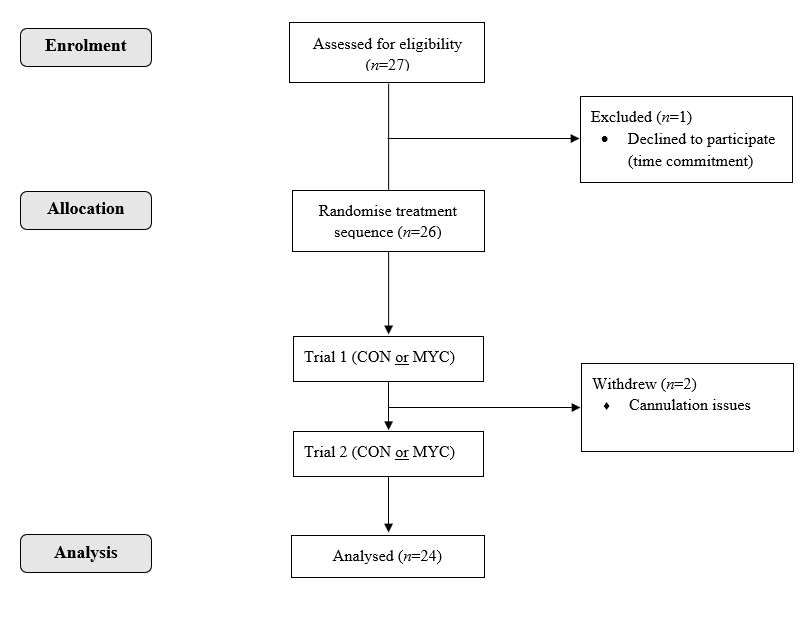


**Figure S1** Participant Flow Diagram





**Figure S2** Glucose kinetics using 100mg ^13^C tracer. Rate of appearance of exogenous glucose (RaEx) and endogenous glucose production (EGP) are shown in graphs A and B.





**Figure S3** Unsmoothed kinetics data following ingestion of the control (CON) and mycoprotein 20 g (MYC). Total rate of glucose appearance (RaT), total rate of disappearance (RdT), total rate of appearance of exogenous glucose (RaEx) and total rate endogenous glucose production (EGP) are shown over 6 h in graphs A, B, C and D. Gridline depicts mean baseline (fasting) values.

**TABLE S1** Respiratory Quotient Values

|  | CON | MYC |
| --- | --- | --- |
| Baseline | 0.83 (0.04) | 0.84 (0.05) |
| 1-hour | 0.86 (0.05) | 0.87 (0.05) |
| 2-hour | 0.85 (0.07) | 0.87 (0.06) |
| 3-hour | 0.85 (0.07) | 0.87 (0.06) |
| 4-hour | 0.83 (0.06) | 0.84 (0.06) |
| 5-hour | 0.81 (0.07) | 0.83 (0.06) |
| 6-hour | 0.81 (0.06) | 0.81 (0.06) |

*Data presented as mean (SD).*

*Values represent the RQ at baseline and at each subsequent hour after ingestion of the control (CON) and mycoprotein (MYC) drink.*

*Two-way ANOVA revealed an effect of time (P<0.0001) but no condition (P=0.38) or interaction effect (time x condition P=0.79).*

1. Debodo, R.C., et al., *On the hormonal regulation of carbohydrate metabolism; studies with C14 glucose.* Recent Prog Horm Res, 1963. **19**: p. 445-88.

2. Livesey, G., et al., *Simultaneous time-varying systemic appearance of oral and hepatic glucose in adults monitored with stable isotopes.* American Journal of Physiology-Endocrinology And Metabolism, 1998. **275**(4): p. E717-E728.

3. Steele, R., et al., *Measurement of size and turnover rate of body glucose pool by the isotope dilution method.* American Journal of Physiology-Legacy Content, 1956. **187**(1): p. 15-24.

4. Wolfe, R.R. and D.L. Chinkes, *Isotope tracers in metabolic research: principles and practice of kinetic analysis*. 2004: John Wiley & Sons.

5. Matsuda, M. and R.A. DeFronzo, *Insulin sensitivity indices obtained from oral glucose tolerance testing: comparison with the euglycemic insulin clamp.* Diabetes care, 1999. **22**(9): p. 1462-1470.

6. Cederholm, J. and L. Wibell, *Insulin release and peripheral sensitivity at the oral glucose tolerance test.* Diabetes research and clinical practice, 1990. **10**(2): p. 167-175.

7. Seltzer, H.S., et al., *Insulin secretion in response to glycemic stimulus: relation of delayed initial release to carbohydrate intolerance in mild diabetes mellitus.* The Journal of clinical investigation, 1967. **46**(3): p. 323-335.

8. Bergman, R.N., D.T. Finegood, and M. Ader, *Assessment of insulin sensitivity in vivo.* Endocrine reviews, 1985. **6**(1): p. 45-86.

9. Frayn, K.N., *Calculation of substrate oxidation rates in vivo from gaseous exchange.* Journal of applied physiology, 1983. **55**(2): p. 628-634.

10. Weir, J.B.d.V., *New methods for calculating metabolic rate with special reference to protein metabolism.* The Journal of physiology, 1949. **109**(1-2): p. 1-9.
